# Histone deacetylase inhibitor CG200745 ameliorates high-fat diet-induced hypertension via inhibition of angiotensin II production

**DOI:** 10.1007/s00210-019-01749-5

**Published:** 2019-10-26

**Authors:** Ga-Eun Yoon, Jin Ki Jung, Yun-Han Lee, Byeong-Churl Jang, Jee In Kim

**Affiliations:** 1 Department of Molecular Medicine and Medical Research Center, Keimyung University School of Medicine, 1095 Dalgubeol-daero, Dalseo-gu, Daegu, 42601 Republic of Korea; 2 Department of Molecular Medicine, Keimyung University School of Medicine, Daegu, Republic of Korea

**Keywords:** HDAC inhibitor, Obesity, Hypertension, Ang II, HDAC activity

## Abstract

Obesity is growing rapidly worldwide due to consumption of westernized diet and lack of exercise. Obesity is one of the major risk factors of hypertension. The novel histone deacetylase (HDAC) inhibitor CG200745 was originally developed to treat various cancers. Previous studies showed that CG200745 attenuated hypertension through inhibition of cardiac hypertrophy and fibrosis in deoxycorticosterone acetate-induced hypertensive rat. The purpose of this study is to investigate the role and underlying mechanism of CG200745 in high-fat diet (HFD)-induced hypertension. Nine-week old C57BL/6 mice were fed a normal diet (ND) or HFD for 17 weeks. Each group of mice was treated with vehicle or CG200745 by intraperitoneal injection for 9 days. HFD group showed higher body weight, blood pressure (BP), HDAC activities, angiotensinogen and renin expressions in kidney, angiotensin-converting enzyme (ACE) expression in the lung, serum angiotensin II (Ang II) concentration, and myosin light chain_20_ (MLC_20_) phosphorylation in mesenteric artery compared with ND group. CG200745 lowered BP, HDAC activity, renin and angiotensinogen in the kidney, ACE in the lung, serum Ang II level, and phosphorylation of MLC_20_ in HFD group. In conclusion, CG200745 ameliorated HFD-induced hypertension through inhibition of HDAC/Ang II/vascular contraction axis. Our results offer CG200745 as a novel therapeutic option for HFD-induced hypertension.

## Introduction

The obese and overweight population is increasing rapidly worldwide due to excessive energy intake from a western diet and lack of physical activity (Popkin and Gordon-Larsen 2004). In 2016, 39% adults of 18 years and older were overweight, and 13% were obese (World Health Organization 2018). If current trends continue, by 2030, up to 57.8% of the adult population could be either of overweight or obese (Kelly et al. 2005). Obesity/overweight often accompanies disorders including diabetes, liver and kidney disease, and cardiovascular disease (Pi-Sunyer 2009), and is one of the major risk factors for hypertension (Mark et al. 1999).

Obesity is known to induce hypertension through activating renin-angiotensin-aldosterone system in the kidney (Thethi et al. 2012). Angiotensin II (Ang II) induces vasoconstriction and consequent blood pressure elevation (Satou et al. 2018). Our previous study showed that increased basal myosin light chain 20 (MLC_20_) phosphorylation and consequent vascular smooth muscle hyper-contractility by enhanced Ang II in high-fat diets (HFD) induced hypertension in rat (Kim 2017). Vascular contraction/relaxation is controlled by various pathways that are calcium-dependent and -independent (Touyz et al. 2018). In aorta smooth muscle cells, Ang II activated RhoA, a member of the Rho family of small GTPase-binding proteins (Seko et al. 2003). Ca^2+^-independent chronic activation of RhoA-activated coiled coil kinase (ROCK) phosphorylates myosin phosphatase target subunit1 (MYPT1) of myosin light chain phosphatase (MLCP) resulting in reduced activity of MLCP and consequent MLC_20_ phosphorylation and vascular contraction (Wirth 2010).

Even though inhibitors for the production of Ang II such as angiotensin-converting enzyme (ACE) inhibitors and Ang II receptor blockers (ARBs) are currently used to treat hypertension (Food and Drug Administration 2011), a variety of side effects limit the usage particularly in obesity-induced hypertension, which demonstrates higher drug resistance (Setaro and Black 1992). Thus, more specific and effective inhibitor is needed to treat obesity-mediated hypertension (Delaney 2009).

Epigenetics suggests new approaches for several diseases including obesity and related disorders (Arguelles et al. 2016). A number of studies showed that histone deacetylase (HDAC) inhibitors are effective to treat cancer, inflammation, fibrosis, and cardiovascular diseases (Tang et al. 2013; Wang et al. 2014). Our previous study reported that pan-HDAC inhibitor valproic acid (VPA) has a protective effect in the HFD-induced hypertension through inhibition of angiotensinogen expression in the kidney (Choi et al. 2017). CG200745 is a recently developed HDAC inhibitor that inhibits class I and class II HDACs (Hyun et al. 2009) and is being tested in phase II clinical trials for its anti-cancer effects (CrystalGenomics 2019). Previous studies showed that CG200745 attenuated hypertension through inhibition of cardiac hypertrophy and fibrosis in deoxycorticosterone acetate (DOCA)-induced hypertensive rat (Lee et al. 2016, 2018) suggesting that CG200745 may also be effective in other hypertension models. Therefore, we investigated the effect and underlying mechanism of CG200745 in HFD-induced hypertension to test the possibility of usage of CG200745 in obesity-mediated hypertension.

Feeding mice HFD recapitulates metabolic, neurohumoral, renal, and cardiovascular alterations observed in obese person (Hall 2003). Thus, the effect and underlying mechanism of CG200745 in high-fat diet-induced hypertension will provide the novel and potent therapeutic option for obese hypertension.

## Materials and methods

### Animal preparation

All animal experiments were conducted in accordance with the guidelines of the National Institutes of Health for the care and use of laboratory animals. The experimental protocol (KM-2017-34R1) was approved by the Institutional Animal Care and Use Committee at Keimyung University. All the ethical regulations were complied. Nine-week-old C57BL/6 male mice (Koatech, Inc., Gyounggido, Korea) were used in this study. Mice were randomly assigned to receive either an HFD containing 60% Kcal from fat (TD.06414, Harlan Laboratories, Inc., Madison, WI, USA) or normal diet (ND) containing 10% Kcal from fat (TD.94048, Harlan Laboratories, Inc., Madison, WI, USA). When the HFD group reached a hypertensive phase, which is over 140 mm Hg systolic blood pressure, mice were administered with CG200745 (0.2 mg kg^−1^ body weight per day by intraperitoneal injection (i.p.); CrystalGenomics, Inc., Gyeonggido, Korea) or vehicle for 9 days.

### Measurement of body weight and consumption of food and water

Body weights were measured using a scale (AND KOREA, Inc., Seoul, Korea) once per week while the mice were fed an ND or HFD. During administration of vehicle or CG200745, body weights were measured daily and consumption of food and water were measured every other day.

### Measurement of blood pressure

Blood pressure was measured using a noninvasive tail-cuff system according to the manufacturer’s instruction. Briefly, mice were preheated on a hot plate at 35 °C for 10 min and then placed in a restrainer. A cuff with a pneumatic pulse sensor was attached to the tail. Blood pressure values were recorded on a CODA High Throughput Noninvasive Blood Pressure system (Kent Scientific, Torrington, CT, USA) on a 35 °C heating pad and were averaged from 10 consecutive readings obtained from each mouse.

### HDAC activity assay

Kidney HDAC activity was determined using HDAC activity fluorometric assay kit (no. K330, BioVision, Inc., Milpitas, CA, USA) according to the manufacturer’s instruction. Briefly, mouse kidney lysate was placed in each black plate well. Ten microliters of 10X HDAC assay buffer and 5 μL of HDAC fluorometric substrate were added to each well, and then the mixture was incubated at room temperature (RT) for 30 min. Ten microliters of Lysine Developer was added, and the excitation/emission at 380/440 nm was detected using a microplate reader (Tecan, Seestrasse, Männedorf, SUI) detecting deacetylated lysin. The HDAC activity was presented as deacetylated lysine (μM/kidney 50 μg) and analyzed using the SigmaPlot (Systat Software, Inc., San Jose, CA, USA).

### Quantitative real-time PCR analysis

RNA was extracted using PureHelix RNA extraction solution (Nanohelix, inc., Daejeon, Korea) from kidney and lung lysate. One microgram of RNA was used for cDNA synthesis using the DiaStar RT Kit (DR23-R10k, SolGent, Inc., Daejeon, Korea). Quantitative real-time PCR (qRT–PCR) was performed using LightCycler 480 SYBR Green I Master mix and the LightCycler machine (Roche Applied Sciences, Basel, Switzerland). Murine qRT–PCR primer sequences were 5′-ACAAACGCATTGCCTGTGAGG AAG-3′ and 5′-TTTGGCTTCTGGCTTCTCCTCCTT-3′ for HDAC1; 5′-TAGGCCTCATAAAGCCACTGCTGA-3′ and 5′-ACCGGACAATCTTCTCCGACGTTA-3′ for HDAC2; 5′-TTCGAGTTCTGCTCCCGTTACACA-3′ and 5′-TAGCAGAAGCCAGAGGCCTCAAAT-3′ for HDAC3; 5′-AACCCTGAGACAAGAGTGCCAGTT-3′ and 5′-TCAGTTGCTCTCTGATGGCATGGA-3′ for HDAC6; 5′-CTCGAACTCAAAGCAGGAGAG-3′ and 5′-GTAGATGGCGAACAGG-AAGG-3′ for angiotensinogen; 5′-GAACGAATCCCGCTC-AAGA A-3′ and 5′-AGGAAGGCCTCTTTGTGAATAC-3′ for renin; 5′-GACAGGTT-CGTGGAAGAGTATG-3′ and 5′-TTGCTGCCCTCTATGGTAATG-3′ for ACE; 5′-GTAACCCGTTGAACCCCATT-3′ and 5′-CCATCCAATCGGTAGTA-GCG-3′ for 18S rRNA, sense and antisense, respectively.

### Ang II enzyme immunoassay

Serum Ang II concentration was measured using Ang II enzyme immunoassay kit (EK-002-12, Phoenix Pharmaceutical, Inc., Burlingame, CA, USA) following the manufacturer’s instruction. Briefly, 50 μL of mouse serum was mixed with 25 μL of primary antibody, 25 μL of biotinylated peptide in the immunoplate precoated with secondary antibody. The mixture was incubated at RT for 2 h. After incubating, the plate was washed, and then 100 μL of streptavidin-horseradish peroxidase solution was added and the mixture was incubated at RT for 1 h. After washing the plate, 100 μL of the TMB (3,3′,5,5′-tetramethylbenzidine) substrate was added and incubated at RT for 1 h. To terminate reaction, 100 μL of 2 N HCl was added, and the optical density at 450 nm was detected using a microplate reader (Bio-Rad, Hercules, CA, USA). The Serum Ang II concentration was analyzed using the SigmaPlot (Systat Software, Inc., San Jose, CA, USA).

### Western blot analysis

Protein samples were prepared by lysis of the kidney and mesenteric artery with lysis buffer containing 5 mM HEPES (pH 7.4), 5 mM EGTA, 1 mM Na_3_VO_4_, 1% Triton X-100, 10% Glycerol, 1 mM DTT, 5 mM NaF, 1 mM PMSF, 5 μg/mL Leupeptin, 2 μg/mL Aprotinin, and 1% sodium deoxycholate. Lysate was centrifuged at 13,000 rpm at 4 °C. Supernatant was taken and the concentration of proteins was measured using BCA kit (23225, Thermo Fisher Scientific, San Diego, CA, USA). Lysates mixed with 5X SDS-PAGE Loading Buffer containing 60 mM tris-HCl (pH 6.8), 50% glycerol, 2% SDS, 0.1% BPB, and 5% 2-mercaptoetanol, and heated at 98 °C for 5 min. SDS samples were stored at − 20 °C until use. Protein samples were electrophoresed on 8~15% polyacrylamide gel with 0.1% SDS and transferred to PVDF or NC membranes, and then subjected to an immunoblotting with antibodies against angiotensinogen (orb10088, Biorbyt, Cambridge, UK), p-MYPT1 at Thr853 (sc-17432 Santa Cruz Biotechnology, Inc., Dallas, TX, USA), p-MLC at Thr18 and Ser19 (PA5-17727, Thermo Fisher Scientific, San Diego, CA, USA), and beta-actin (sc-47778, Santa Cruz Biotechnology, Inc., Dallas, Texas, USA). Horseradish peroxidase-conjugated secondary antibodies (anti-rabbit: A120-101P, bethyl laboratories, Inc., montgomery, TX, USA; anti-goat: A50-101P, bethyl laboratories, Inc., montgomery, TX, USA; anti-mouse: A90-116P, bethyl laboratories, Inc., montgomery, TX, USA) were applied, and immunoblots were visualized using chemiluminescence reagent (NEL104001EA, PerkinElmer Life Sciences, Inc., Waltham, MA, USA). Densities of immunoblots were quantified using image analysis software ImageJ (National Institutes of Health, Inc., Bethesda, ML, USA).

### Statistical analysis

The results are expressed as the mean ± SE. Statistical significance were evaluated with an analysis of variance using Student’s *t* test. Differences between groups were considered statistically significant with a *p* value of < 0.05.

## Results

### HFD increased body weight and blood pressure

**Fig. 1:**
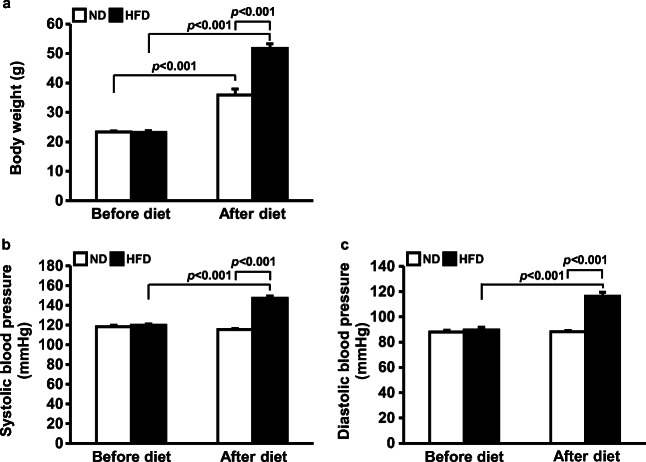
Increased body weight and blood pressure by HFD. Mice were fed either ND or HFD for 17 weeks. Blood pressure was measured using the tail-cuff method. Graphs summarize body weight (**a**), systolic blood pressure (**b**), and diastolic blood pressure (**c**). HFD accelerated increase in body weight and blood pressure. Results are expressed as the mean ± SE (*n* = 5–8 mice per group). ND, normal diet; HFD, high-fat diet

To induce obesity-mediated hypertension, mice were randomly divided into two groups and fed a ND or HFD for 17 weeks. Before feeding different diets, there was no difference between the body weights of the two groups. After 17 weeks of consuming different diets, both groups showed increased body weights, while HFD significantly accelerated the body weight increase (from 22.8 ± 0.2 to 34.7 ± 0.9 g in the ND group, from 23.0 ± 0.3 to 48.1 ± 1.4 g in the HFD group) (*p* < 0.001 ND vs. HFD) (Fig. 1a). Before feeding different diets, there was no difference between the systolic blood pressures of the two groups. The ND did not affect systolic blood pressures (from 118.4 ± 1.4 to 115.4 ± 1.2 mm Hg), but the HFD significantly increased the systolic blood pressures (from 119.8 ± 1.3 to 147.3 ± 2.2 mm Hg) (*p* < 0.001 before HFD vs. after HFD) (Fig. 1b). The diastolic blood pressures were also not different between groups before feeding different diets. The ND did not affect diastolic blood pressure (from 88.0 ± 1.3 to 88.2 ± 1.0 mm Hg), but the HFD increased diastolic blood pressure (from 89.6 ± 2.3 to 116.3 ± 3.2 mm Hg) (*p* < 0.001 before HFD vs. after HFD) (Fig. 1c).

### Treatment of CG200745 ameliorated HFD-induced hypertension

**Fig. 2:**
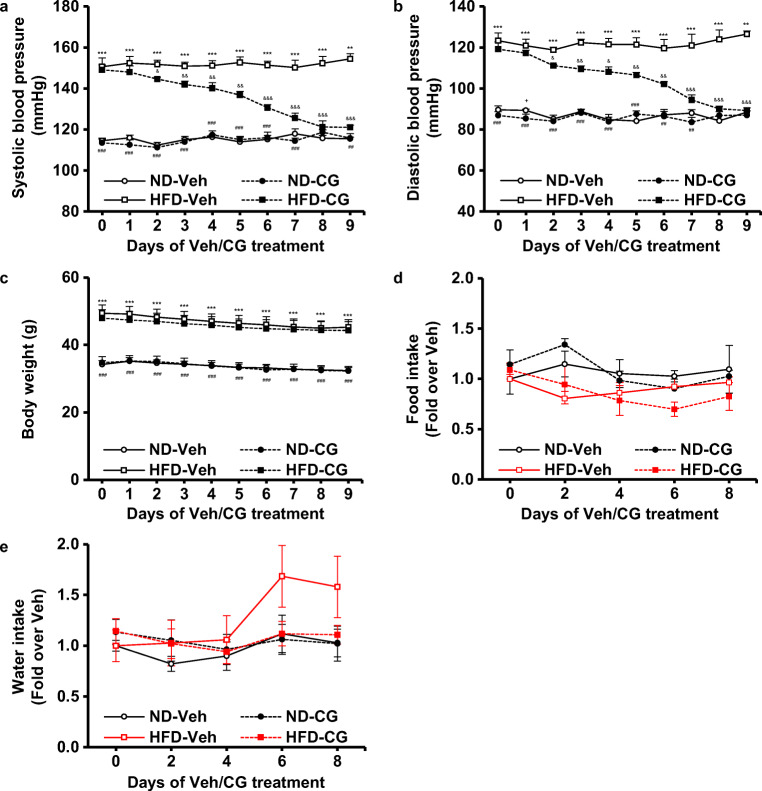
Blood pressure and body weight after treatment of CG200745 in ND- and HFD-fed mice. Graphs summarize systolic blood pressure (**a**), diastolic blood pressure (**b**), body weight (**c**), food intake (**d**), and water intake (**e**) in groups of ND with vehicle, ND with CG, HFD with vehicle, and HFD with CG. Treatment with CG lowered systolic and diastolic blood pressure in the HFD-fed group gradually but did not affect body weight and consumption of food and water. (**p < 0.01, ***p < 0.001 vehicle-HFD vs. ND; ^##^*p* < 0.01, ^###^*p* < 0.001 CG-HFD vs. ND; ^+^*p* < 0.05 CG-ND vs. vehicle-ND; ^&^*p* < 0.05, ^&&^*p* < 0.01, ^&&&^*p* < 0.001 CG-HFD vs. vehicle-HFD). Results are expressed as the mean ± SE (*n* = 5–8 mice per group). ND, normal diet; HFD, high-fat diet; Veh, vehicle; CG, CG200745

To investigate the effect of CG200745 on HFD-induced hypertension, each diet-fed group was administered with vehicle or CG200745 (0.2 mg kg^−1^ day^−1^, i.p.). The blood pressure of ND-fed mice did not change in response to either vehicle or CG200745 (Fig. 2a, b). While the vehicle-administered HFD group maintained high systolic and diastolic blood pressure, the CG200745 treatment lowered the blood pressure to the normal state (from 149.1 ± 2.5 to 121.0 ± 1.2 mm Hg of systolic blood pressure, from 119.2 ± 3.5 to 89.3 ± 1.2 mm Hg of diastolic blood pressure) (*p* < 0.001 vehicle-HFD vs. CG200745-HFD for both systolic and diastolic blood pressures) (Fig. 2a, b). The body weights and consumption of food and water of ND- and HFD-fed mice did not change with the CG200745 treatment (Fig. 2c–e).

### CG200745 reversed HFD-induced increase in HDAC activity and expression in mouse kidney

**Fig. 3:**
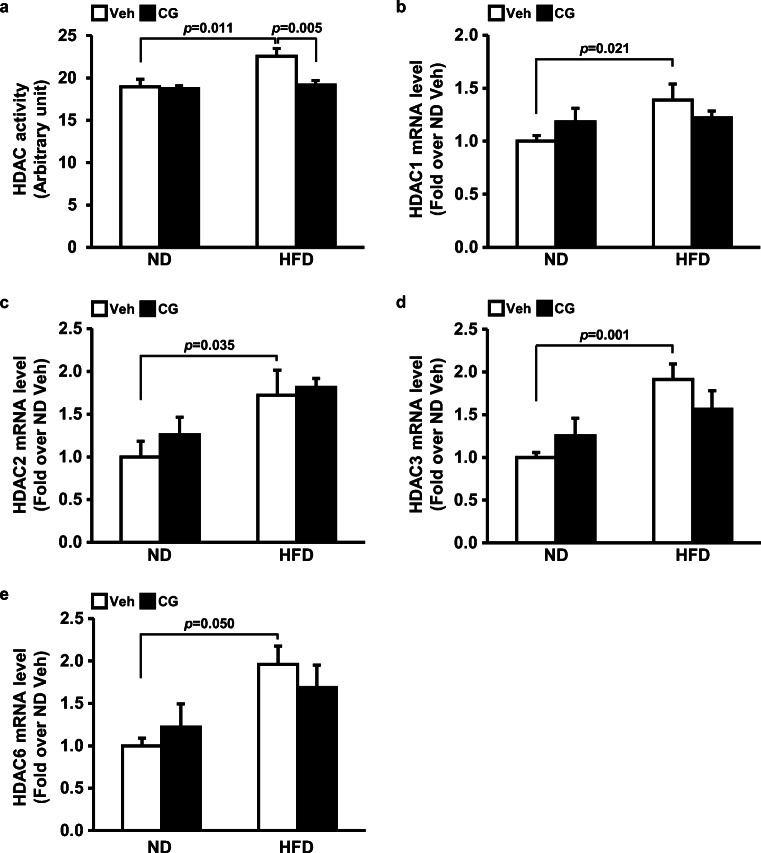
Effect of CG200745 on HDAC activity in the kidney of ND- and HFD-fed mice. Graphs summarize kidney HDAC activity and mRNA expressions of HDAC 1, 2, 3, and 6. HDAC activities were increased by HFD and reversed by CG200745 to the ND level. mRNA expressions of HDAC 1, 2, 3, and 6 were increased by HFD but not decreased by CG200745. Results are expressed as the mean ± SE (*n* = 5–8 mice per group). HDAC, histone deacetylase; ND, normal diet; HFD, high-fat diet; Veh, vehicle; CG, CG200745

HDAC activity in mouse kidney was higher in HFD group (22.6 ± 0.9 μM/kidney 50 μg) than in ND group (19.0 ± 0.9 μM/kidney 50 μg) (*p* = 0.011 vehicle-ND vs. vehicle-HFD). The CG200745 treatment to HFD group reversed the HDAC activity (19.1 ± 0.6 μM/kidney 50 μg) to the level in the ND group (*p* = 0.005 vehicle-HFD vs. CG200745-HFD). CG200745 did not affect HDAC activity in the ND group (Fig. 3a). mRNA expressions of HDAC1, 2, 3, and 6 in the kidney were higher in HFD group than in ND group. However, CG200745 administration did not significantly downregulated the expressions of HDACs in the HFD group (Fig. 3b–e) indicating that 200 μg/kg of CG200745 could reduce HDACs activities but not expressions.

### CG200745 downregulated the expressions of renin-angiotensin system components and serum Ang II in HFD-fed group

**Fig. 4:**
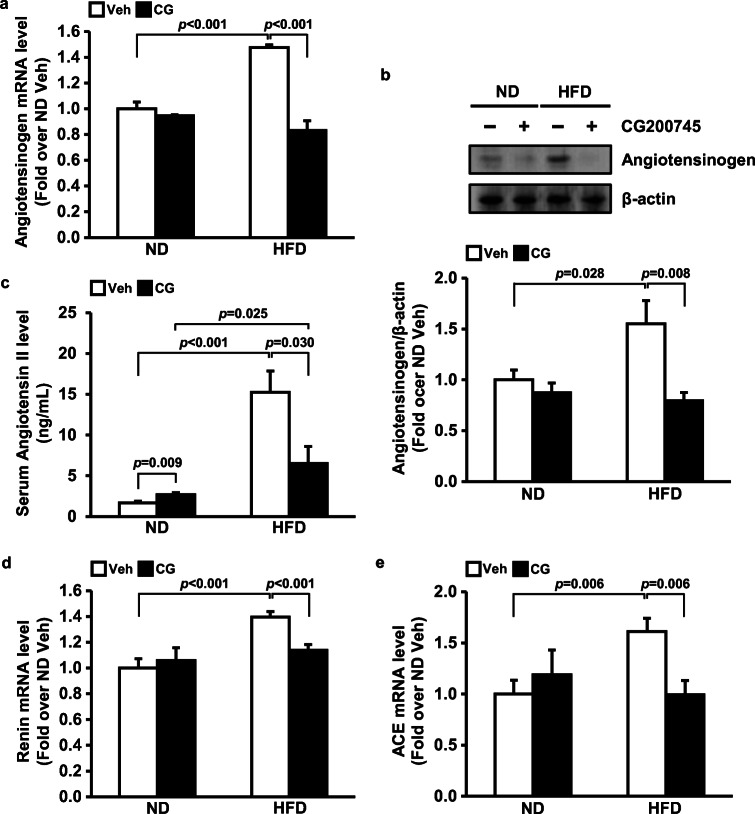
Effect of CG200745 on renin-angiotensin system in the ND- or HFD-fed mice. Graphs summarize kidney mRNA expression of angiotensinogen (**a**), protein expression of angiotensinogen and representative picture of western blot (**b**), serum concentration of Ang II (**c**), kidney mRNA expression of renin (**d**), lung mRNA expression of ACE (**e**). **a–e** Renin-angiotensin system components were increased by HFD and reversed to the normal level by CG200745 treatment. Results are expressed as the mean ± SE (*n* = 3–5 mice per group). ND, normal diet; HFD, high-fat diet; Veh, vehicle; CG, CG200745; Ang II, angiotensin II; ACE, angiotensin-converting enzyme

mRNA expression of angiotensinogen in the kidney was higher in HFD group (1.5 ± 0.0-fold) than in ND group (1.0 ± 0.1-fold) (*p* < 0.001 vehicle-ND vs. vehicle-HFD). CG200745 administration downregulated the expression of angiotensinogen in the HFD group to the ND group level (0.8 ± 0.1-fold) (*p* < 0.001 vehicle-HFD vs. CG200745-HFD) (Fig. 4a). Protein angiotensinogen level was also upregulated in HFD group in the kidney (1.6 ± 0.2-fold) than in ND group (1.0 ± 0.1-fold) (*p* = 0.028 vehicle-ND vs. vehicle-HFD). CG200745 administration downregulated the protein angiotensinogen level in the HFD group (0.8 ± 0.1-fold) (*p* = 0.008 vehicle-HFD vs. CG200745-HFD) (Fig. 4b). Serum Ang II concentration was higher in the HFD group (15.2 ± 2.6 ng/μL) than in the ND group (1.7 ± 0.2 ng/μL) (*p* < 0.001 vehicle-ND vs. vehicle-HFD). CG200745 administration decreased serum Ang II (6.5 ± 2.1 ng/μL) in the HFD-fed mice (*p* = 0.030 vehicle-HFD vs. CG200745-HFD) (Fig. 4c). mRNA expression of renin in the kidney was higher in the HFD group (1.4 ± 0.0-fold) than in ND group (1.0 ± 0.1-fold) (*p* < 0.001 vehicle-ND vs. vehicle-HFD). CG200745 administration downregulated the expression of renin in the HFD group to the ND group level (1.1 ± 0.0-fold) (*p* = 0.001 vehicle-HFD vs. CG200745-HFD) (Fig. 4d). mRNA expression of ACE in the lung was higher in the HFD group (1.6 ± 0.1-fold) than in ND group (1.0 ± 0.1-fold) (*p* = 0.006 vehicle-ND vs. vehicle-HFD). CG200745 administration downregulated the expression of ACE in the HFD group to the ND group level (1.0 ± 0.1-fold) (*p* = 0.006 vehicle-HFD vs. CG200745-HFD) (Fig. 4e).

### CG200745 decreased the phosphorylation of MLC_20_ via phosphorylation of MYPT1 in mouse mesenteric artery

**Fig. 5:**
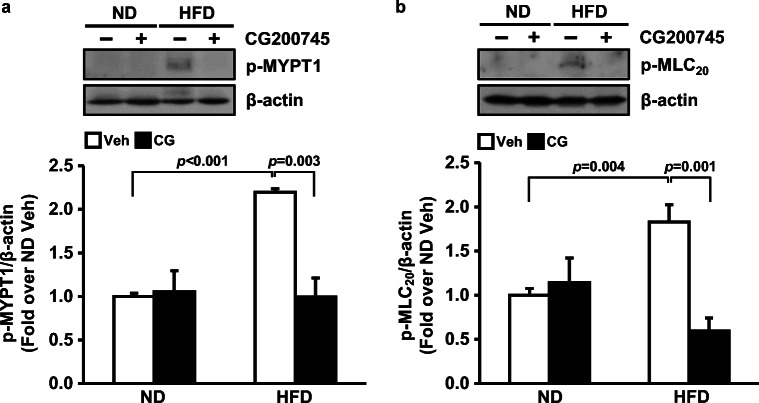
Effect of CG200745 on vascular contraction. Representative picture of western blot for phosphorylated MLC_20_ at Thr18 and Ser19, phosphorylated MYPT1 at Thr853, and internal control beta-actin. Graphs summarize p-MYPT1 (**a**) and p-MLC_20_ (**b**) in the mesenteric artery of ND and HFD-fed mice with/without CG200745 treatment. p-MYPT1 and p-MLC_20_ were increased by HFD. CG200745 decreased phosphorylation of MYPT1 and MLC_20_. Data are presented as the mean ± SE (*n* = 3–5 mice per group). p-, phosphorylated; MYPT1, Myosin phosphatase-targeting subunit1; MLC_20_, myosin light chain 20; ND, normal diet; HFD, high-fat diet; Veh, vehicle; CG, CG200745

To investigate the mechanism by which HFD-induced hypertension was ameliorated by CG200745, we measured phosphorylation of MYPT1, which increases phosphorylation of MLC_20_ resulting in vascular contraction. MYPT1 phosphorylation was higher in the HFD group (2.2 ± 0.0-fold) than in ND group (1.0 ± 0.0-fold) (*p* < 0.001 vehicle-ND vs. vehicle-HFD). MLC_20_ phosphorylation was higher in the HFD group (1.8 ± 0.2-fold) than in ND group (1.0 ± 0.1-fold) (*p* = 0.004 vehicle-ND vs. vehicle-HFD). CG200745 administration downregulated the phosphorylation of MYPT1 in the HFD group to the ND group level (1.0 ± 0.2-fold) (*p* = 0.003 vehicle-HFD vs. CG200745-HFD). CG200745 administration downregulated the phosphorylation of MLC_20_ in the HFD group (0.6 ± 0.1-fold) (*p* = 0.001 vehicle-HFD vs. CG200745-HFD) (Fig. 5a, b).

## Discussion

The most important finding of the current study is that novel HDAC inhibitor CG200745, which was developed for cancer treatment, is useful for ameliorating HFD-induced hypertension and the major target is Ang II. These data offer CG200745 as a novel therapeutic option for the obese hypertension.

HDAC inhibitors have been studied primarily in the field of cancer and recently studied in cardiovascular diseases (Yoon and Eom 2016; Eckschlager et al. 2017). Various HDAC inhibitors have shown anti-hypertensive effects through various mechanisms. Although it has been generally accepted that acetylation of histones loosens the chromosomes and recruits transcription factors to the promoter region resulting in activation of gene expression, recent studies showed that this is not always the case. For example, VPA-induced histone acetylation suppresses CYP19 gene expression (Chen et al. 2015). VPA suppressed adiponectin gene expression by reducing adipogenic transcription factor C/EBPα level and binding of C/EBPα to the adiponectin promoter (Qiao et al. 2006). We also revealed downregulation of angiotensinogen, renin, and ACE in this study and our previous study (Choi et al. 2017). Further, HDACs deacetylate not only histones but also other proteins, including transcription factors to regulate the transcriptional activity resulting in alteration of gene expressions. VPA prevented hypertension via acetylation-induced transcriptional inactivation of mineralocorticoid receptor, suppressing many target genes of mineralocorticoid receptor in DOCA-induced hypertensive rats (Lee et al. 2013). In addition, MS-275, class I HDAC-selective inhibitor, has been reported to have anti-hypertensive effect to reduce blood vessel thickness and to inhibit inflammation in an Ang II-induced hypertensive animal model (Ryu et al. 2019). LMK235, class I and HDAC6-preferential HDAC inhibitor, ameliorated hypertension via inhibition of vascular contraction and vessel hypertrophy in mouse Ang II-infusion model and spontaneously hypertensive rat model (Choi et al. 2019).

Our previous and present studies show increased vascular smooth muscle contractility by increased Ang II in HFD-induced hypertension with increased phosphorylation of MYPT1 of MLCP and MLC_20_ (Kim 2017). Here we revealed that CG200745 reversed the increase in serum Ang II and vascular contraction with decreased phosphorylation of MYPT1 and MLC_20_. Our results indicate that this happened via inhibition of HFD/HDACs/Ang II/deactivation of MLCP/MLC_20_ phosphorylation-vascular contraction axis. So far, there is no evidence that HDACs directly inhibit MLCP.

The expressions of HDACs are regulated tissue- and disease-dependently. For example, even though we showed that HDACs were upregulated in the HFD-fed mouse kidneys, expressions of HDAC1, 2, and 3 were fluctuated time-dependently but mostly reduced in lungs from mice with cecal ligation and puncture (CLP)-induced sepsis. CG200745 treatment protected septic mice from lung and splenic apoptosis (Takebe et al. 2014).

Our previous study showed that VPA prevented HFD-induced hypertension by downregulating angiotensinogen and its receptor in the kidney. That demonstrated increased HDAC1 expression and decreased histone acetylation suggesting increased HDAC activity indirectly in the HFD-induced hypertension (Choi et al. 2017). Meanwhile, in this study, we revealed the increased kidney HDAC activity directly in HFD-induced hypertension using assay to measure deacetylating capability of HDAC in the tissue lysate. CG200745 is a novel HDAC inhibitor that is in phase II clinical trial for anti-cancer effect (CrystalGenomics 2019). CG200745 ameliorated DOCA-induced hypertension through inhibition of cardiac hypertrophy and fibrosis (Lee et al. 2016) and renoprotective action (Bae et al. 2019). Here, we revealed that CG200745 reversed HFD-induced increase in HDAC activity, kidney angiotensinogen expression, and consequent increase in serum Ang II resulting in vascular contraction and amelioration of HFD-induced hypertension for the first time.

The Ang II is one of the critical mediators to induce hypertension including obese hypertension (Sparks et al. 2014). Even though it is generally accepted that liver is the main organ to produce angiotensinogen, it is also reported to be produced in various tissues including the kidney, brain, spinal cord, aorta, mesentery, atrium, lung, adrenal gland, large intestine, stomach and spleen (Campbell and Habener 1986). Interestingly, even though the kidney is not the main organ to produce angiotensinogen, increased production of angiotensinogen in the kidney became critical factor to induce the hypertension in model of type 2 diabetes mellitus (Woods et al. 2019). Our previous and the present study also revealed increased angiotensinogen in the kidney and consequent increase in Ang II in the plasma in HFD-induced hypertension proving importance of renal angiotensinogen expression (Choi et al. 2017; Kim 2017).

Ang II inhibitors such as ACE inhibitors and ARBs have developed as anti-hypertensive agents and used in patients with hypertension. However, many side effects including cough, dizziness, fatigue, headache, sleeping disorder, tachycardia, sore throat, sinus problems, heartburn, diarrhea, and back pain have been reported limiting its usage (Food and Drug Administration 2011). Unfortunately, obese hypertension patients are often more resistant to anti-hypertensive drugs (Chobanian 2009). Therefore, development of specific inhibitor for Ang II will provide better therapeutics for obese hypertension. The common side effects reported in patients who have been prescribed ACE inhibitors are caused by the accumulation of inflammatory compounds such as bradykinin and substance P, the release of which is stimulated by ACE inhibitors (Devin et al. 2014; Scalese and Reinaker 2016). The most severe symptom associated with ACE inhibitors angioedema occurs in 0.55–1.62% of patients according to the Octave study (Kostis et al. 2004). Airway swelling and obstruction due to the accumulation of fluid and bradykinin are the main features of angioedema (Scalese and Reinaker 2016). Patients experiencing angioedema while using an ACE inhibitor must discontinue the medication and avoid all ACE inhibitors in the future. ACE inhibitor-associated anemia is due to the suppression of erythropoietin production, in response to A-acetyl-seryl-aspartyl-lysyl-proline accumulation in plasma (Yildiz et al. 2001). We think CG200745 will not share side effects with ACE inhibitors because accumulation of bradykinin, substance P, and A-acetyl-seryl-aspartyl-lysyl-proline is unique phenomenon of ACE inhibitors. Functional renal insufficiency occurs by ACE inhibitors and ARBs. It happens due to fall in glomerular afferent arteriolar flow and consequent vasodilatation of glomerular efferent arteriole via inhibition of Ang II action. Functional renal insufficiency occurs in patients with severe renal artery stenosis, solitary kidney, dehydration, use of NSAIDs, heart failure, and microvascular disease (Schoolwerth et al. 2001). We presume that CG200745 may not cause renal insufficiency because CG200745 works by inhibiting over-production of angiotensinogen or Ang II in response to HFD not by random inhibition of existing angiotensinogen or Ang II. Of course the side effects of CG200745 should be studied thoroughly but good thing is that the dose we used in this study (200 μg/ kg) is greatly lower than the dose of clinical trial (1.275–6.25 mg/kg) (Kim et al. 2015) or other researches at 5 mg/kg (Lee et al. 2016; Bae et al. 2019) or 35–45 mg/kg (Hwang et al. 2012; Jung et al. 2017) reducing the possibility of side effects. In this study, even combined with HFD, 200 μg/kg i.p. of CG200745 dropped the blood pressure to the normal level after 6 days of treatment with the decreased Ang II level. Therefore, we offer novel HDAC inhibitor CG200745 as a potent therapeutic option for HFD-induced hypertension via inhibition of Ang II.
